# Evaluation of Proton-Induced DNA Damage in 3D-Engineered Glioblastoma Microenvironments

**DOI:** 10.1021/acsami.2c03706

**Published:** 2022-04-20

**Authors:** Qais Akolawala, Marta Rovituso, Henri H. Versteeg, Araci M. R. Rondon, Angelo Accardo

**Affiliations:** † Department of Precision and Microsystems Engineering, Delft University of Technology, Mekelweg 2, 2628 CD Delft, The Netherlands; ‡ Holland Proton Therapy Center (HollandPTC), Huismansingel 4, 2629 JH Delft, The Netherlands; § Einthoven Laboratory for Vascular and Regenerative Medicine, Division of Thrombosis and Hemostasis, Department of Internal Medicine, Leiden University Medical Center, 2333 ZA Leiden, The Netherlands

**Keywords:** engineered cell microenvironments, two-photon polymerization, cancer, glioblastoma, proton therapy, DNA damage

## Abstract

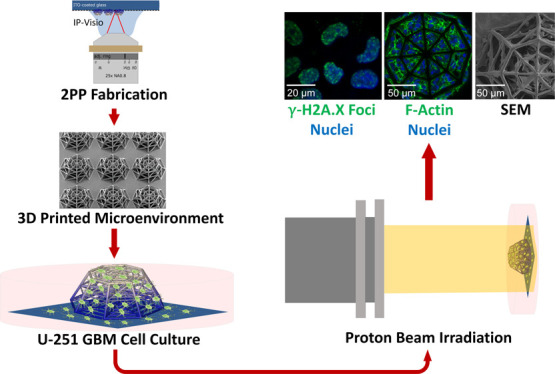

Glioblastoma (GBM) is a devastating cancer of the brain with an extremely poor prognosis. For this reason, besides clinical and preclinical studies, novel *in vitro* models for the assessment of cancer response to drugs and radiation are being developed. In such context, three-dimensional (3D)-engineered cellular microenvironments, compared to unrealistic two-dimensional (2D) monolayer cell culture, provide a model closer to the *in vivo* configuration. Concerning cancer treatment, while X-ray radiotherapy and chemotherapy remain the current standard, proton beam therapy is an appealing alternative as protons can be efficiently targeted to destroy cancer cells while sparing the surrounding healthy tissue. However, despite the treatment’s compelling biological and medical rationale, little is known about the effects of protons on GBM at the cellular level. In this work, we designed novel 3D-engineered scaffolds inspired by the geometry of brain blood vessels, which cover a vital role in the colonization mechanisms of GBM cells. The architectures were fabricated by two-photon polymerization (2PP), cultured with U-251 GBM cells and integrated for the first time in the context of proton radiation experiments to assess their response to treatment. We employed Gamma H2A.X as a fluorescent biomarker to identify the DNA damage induced in the cells by proton beams. The results show a higher DNA double-strand breakage in 2D cell monolayers as compared to cells cultured in 3D. The discrepancy in terms of proton radiation response could indicate a difference in the radioresistance of the GBM cells or in the rate of repair kinetics between 2D cell monolayers and 3D cell networks. Thus, these biomimetic-engineered 3D scaffolds pave the way for the realization of a benchmark tool that can be used to routinely assess the effects of proton therapy on 3D GBM cell networks and other types of cancer cells.

## 1 Introduction

The development of three-dimensional (3D)-engineered cell culture models^1,2^ over conventional two-dimensional (2D) “Petri dish” approaches featured a rapid expansion during the last decade. The main reason is that 2D approaches lead to the formation of unrealistic cell monolayers, while the first ones enable cells to grow within a 3D spatial configuration closer to the *in vivo* tissue architecture. 3D cell culture models are often categorized into scaffold-free approaches and scaffold-based ones.^3,4^ The first ones include a family of cell self-assembly techniques leading to the formation of multicellular 3D tissue-like structures called spheroids and organoids, which facilitate intense cell–cell interactions and resemble physiological conditions of complex tissues via spatially defined differentiation.^5^ The second category comprises a series of additive manufacturing techniques^6,7^ employed to build artificial skeletons, usually made of polymeric or hydrogel material,^8^ called “scaffolds”, where cells can grow along precise 3D structures. Among these techniques, we find fused deposition modeling (FDM), where the 3D object is obtained, layer by layer, by extruding a thermoplastic material through the nozzle of a moving head which displacement is remotely controlled by a computer.^9^ Another technique similar to FDM is called bioprinting (BP).^10^ The difference is that in this case cells are incorporated directly in bioinks (made of hydrogel materials) and extruded under cell-compatible conditions (temperature of 37 °C). While these two techniques hold the great advantage to have access to a wide library of intrinsically biocompatible materials, they are characterized by a very low feature resolution, in the range of hundreds of microns, which is far from the cellular scale. This can be detrimental as it is known that cells are very sensitive to geometries and curvatures with features in the micrometric or even submicrometric range.^11,12^ To overcome this limitation, it is possible to resort to laser-assisted fabrication technologies such as stereolithography (SLA)^13,14^ and two-photon polymerization (2PP).^15,16^ The first technique can reach micrometric resolution and manufacture centimeter-sized objects by exploiting a layer-by-layer approach, where a UV laser source photopolymerizes a series of transverse-plane image slices of a photoresin that finally leads to the realization of a 3D design. The physical mechanism underlying the 2PP fabrication, widely used in the cell mechanobiology field,^15^ exploits two-photon absorption (TPA) of near-infrared radiation (NIR) by focusing femtosecond laser pulses onto an organic prepolymer material highly absorptive in the UV radiation range while “transparent” in the infrared radiation one. This nonlinear mechanism is tuned to induce the photopolymerization of the exposed material in extremely confined volumes called voxels, featuring dimensions in the submicrometric range.

In the field of engineered 3D cell microenvironments, the development of architectures for the study and treatment of cancer cells assumes a paramount importance for fundamental mechanobiology and prospective clinical studies.^17−20^ Among the different types of cancer, glioblastoma (GBM) is a grade IV glioma brain tumor^21^ and the most aggressive type of brain cancer with an incidence of 2–3 cases per 100.000 people per year.^22^ GBM continues to have one of the most dismal prognoses of any cancer with a median survival rate of about 12–15 months and less than 5% of cases surviving over 5 years after initial diagnosis.^23,24^ The current treatment regime includes removal of the bulk of the tumor by surgery followed by image-guided radiotherapy (where high-energy X-ray radiation destroys the cancer cells) and chemotherapy to control the regrowth of the tumor. Nonetheless, GBM has a high propensity of recurrence and therefore often needs multiple surgeries,^25^ which are not always possible (e.g., when the GBM is growing in the deep core of the brain tissue). Chemotherapy has many toxic side effects, and the blood–brain barrier limits drug penetration. Conventional X-ray radiotherapy is known to damage not only cancer cells but also the healthy surrounding brain tissue. A more recent technique trying to tackle cancer, and in particular brain cancer, improving on the drawbacks of conventional radiotherapy, is proton beam therapy (PBT), which employs protons^26^ instead of X-rays. Compared to X-rays, protons can be focused exclusively on the cancerous tissue with much less damage to the healthy tissue. Unfortunately, the higher cost of proton radiotherapy treatments has led to a lack of clinical information, which is the main reason why many questions remain about the efficiency of proton irradiation on GBM at the cellular level.^27,28^ This is mostly because systematic studies on the morphological and functional changes (e.g., DNA damage) of the cells after being exposed cannot be routinely performed on animals, due to their scarcity and ethical reasons, or living tissues derived from biopsies due as well to their scarcity and the difficulty in preserving them alive for a long time.

To fill this knowledge gap, we report for the first time the creation of standardized, reproducible, and physiologically relevant 3D-engineered GBM microenvironments, fabricated by 2PP, to assess the DNA damage of proton beams on brain cancer cells. Indeed, although previous works reported on the effect of charged particles *in vitro* on GBM cells in 2D monolayers^29^ and 3D-collagen matrices,^30^ there are no studies about the use of 3D-engineered 2PP microenvironments to assess the effect of proton beams on GBM 3D cellular networks. In our investigation, we employed a novel photosensitive biomaterial called IP-Visio characterized by high biocompatibility, favoring the growth and adhesion of GBM cells, and negligible intrinsic autofluorescence, enabling immunofluorescence characterization of cell biomarkers. The GBM cell line U-251 was cultured on both 2D IP-Visio pedestals and 3D IP-Visio microenvironments to compare morphological cell features as well as the amount of DNA damage induced by two different proton radiation doses (2 and 8 Gy) in Spread-out Bragg peak (SOBP) configuration,^31^ which enables a homogenous distribution of protons within a constrained volume. While on 2D pedestals we obtained, as expected, the formation of compact cell monolayers with very thin cytoplasmic extensions, the 3D GBM microenvironment fostered cell colonization in three dimensions with the formation of long protrusions that extended across the scaffold owing to the presence of rationally designed geometries. Remarkably, 2D GBM cell monolayers featured a much higher amount of DNA damage, especially for the 8 Gy dose, compared to 3D GBM-engineered microenvironments, showing that 3D GBM cell networks, mimicking more closely the *in vivo* tissue configuration, respond differently to proton radiation doses. This study paves therefore the way for the use of 2PP-engineered GBM 3D microenvironments as a benchmark tool for proton radiobiology, which can be potentially extended also to other cancer cells.

## 2 Experimental Section

### 2.1 Direct-Laser-Writing Setup Configuration

A commercial 2PP setup (Nanoscribe Photonic Professional GT+) was used to manufacture both the 2D pedestals and 3D scaffolds. The 3D scaffolds were first designed by computer-aided design (CAD) software, Autodesk Fusion 360, and then exported to an STL format. The STL file was then elaborated by Nanoscribe DeScribe software to convert it into Nanoscribe’s General Writing Language (GWL). The GWL file was finally provided to the NanoWrite program that controls the 2PP setup. During the conversion process, the STL file is sliced into 2D layers and each of these planes is then transformed into a set of hatched lines. A droplet of commercially available negative tone photoresist, known as IP-Visio (featuring a methacrylate functional group), was cast on cleaned and silanized ITO-coated (indium–tin oxide: thickness 18 ± 5 nm) soda-lime glass substrates (25 mm × 25 mm, 0.7 mm thickness). The substrates were cleaned and activated with O_2_ plasma (Diener Femto plasma etcher) at a power of 80 W for 10 min, O_2_ flow at 5 sccm, and pressure of 0.1 bar. Consequently, they were silanized by immersion for 1 h in a 2% v/v 3-(trimethoxysilyl) propyl methacrylate (MAPTMS, Sigma-Aldrich)/ethanol solution and then rinsed with acetone, water, and dried with a compressed air gun in between rinsing steps. Silanization increases the adhesion of the photosensitive biomaterial with the substrate. The resin was then exposed to a 780 nm wavelength, femtosecond pulsed laser (100 fs, 50 mW corresponding to 100% power intensity) with the Nanoscribe Photonic Professional GT+ two-photon polymerization system through a 25X immersion objective (NA = 0.8), and using a “Galvo” configuration where mirrors scan the laser beam laterally, and the vertical movement is carried out with piezo actuators. This moving beam fixed sample (MBFS) approach was used with the dip-in laser lithography (DiLL) configuration in which the objective is dipped in the photoresist (Figure S1A). The specific parameters used for printing the free-standing 3D structures were 100% laser power and a scanning speed of 15 mm/s. Concerning the 2D pedestals, the optimal parameters were 80% laser power with a 50 mm/s scanning speed. The slicing (distance between adjacent layers) and hatching (lateral distance between adjacent lines) parameters for both 2D and 3D structures were set at 800 and 500 nm, respectively. Each 3D scaffold and 2D pedestal was fabricated in 4 and 10 min, respectively. Overall, each 2PP-printed sample consisted of 15 scaffolds and 3 pedestals (≈90 min printing time). The samples were then chemically developed in propylene glycol monomethyl ether acetate (PGMEA, Sigma-Aldrich) for 25 min, rinsed with 2-propanol (IPA, Sigma-Aldrich) for 5 min, and then air-dried under a chemical fume hood.

### 2.2 GBM Cell Culture

Prior to cell culture, the printed samples were transferred to sterile Petri dishes (60 mm diameter) in a tissue culture hood. Each sample was sterilized by immersion in 70% ethanol for 10 min and subsequently gently washed with phosphate-buffered saline (PBS). The samples were then allowed to air dry. The Human GBM U-251 (U-251 MG, previously known as U-373 MG, ATCC HTB-17) was kindly donated by Prof. Janusz Rak, McGill University, Canada. U-251 cells were cultured using Dulbecco’s modified Eagle’s medium (DMEM, Gibco) with 1% l-glutamine (Sigma-Aldrich), 1% penicillin–streptomycin (P/S, Gibco), and 10% fetal bovine serum (FBS, PAN Biotech). DNA profiling using short tandem repeat markers was performed to confirm the origin of U-251, and mycoplasma testing was performed monthly using MycoAlertTM Mycoplasma Detection Kit (Lonza). Typically, 50,000 cells/mL were inoculated onto the scaffold in droplets of 75 μL around the scaffold region. The cells were allowed to adhere to the scaffolds for 1 h in a cell culture incubator at 37 °C and 5% CO_2_. Five milliliters of DMEM (with 10% FBS, 1%P/S) was gently added to the dishes and left in the incubator for 5 days before characterization or proton irradiation.

### 2.3 Sample Preparation for Scanning Electron Microscopy (SEM) and Immunofluorescence Staining Protocols

To prepare the sample for SEM characterization, cells were rinsed with PBS and incubated in 4% glutaraldehyde (in PBS) solution for 4 h at room temperature. The glutaraldehyde was then removed, and cells were rinsed with PBS. Cells were then incubated in 50, 70, 90, and 100% ethanol for 4 min each and immersed in 33, 50, 66, and 100% solutions of hexamethyldisilazane (HMDS, Sigma-Aldrich) in 100% ethanol for 15 min each. Finally, the residual HMDS was allowed to evaporate overnight. The whole protocol was carried out in a chemical fume hood.

To perform indirect immunofluorescence staining for confocal microscopy, cells were fixed with 4% paraformaldehyde in PBS for 15 min, permeabilized in 0.2% Triton X-100 for 15 min, and nonspecific protein binding sites were blocked with 5% bovine serum albumin (BSA). Cells were incubated with the DNA damage antibody anti-Gamma H2A.X (phospho Serine 139, 1:250 dilution in 1% BSA/PBS, Abcam) for 60 min to detect DNA damage, followed by incubation with a FITC-conjugated goat antirabbit IgG secondary antibody (1:500 dilution in 1% BSA, Molecular Probes) for 60 min at room temperature in a humid chamber. The nuclei were stained with Hoechst 33258 (1:1000 dilution, Molecular Probes) for 5 min. After the staining, cells were imaged or stored in PBS at 4 °C. Concerning cell morphology visualization via direct immunofluorescence, the cells were fixed with 4% paraformaldehyde in PBS for 15 min and permeabilized in 0.1% Triton X-100 (Merck) for 15 min. The cells were stained with a 50 μg/mL fluorescent phalloidin–FITC conjugate (Sigma-Aldrich) solution in PBS for 40 min at room temperature to visualize F-actin in the cellular cytoskeleton. The cells were washed, and the nuclei were counterstained with Hoechst 33258 (1:1000 dilution, Molecular Probes) for 5 min.

### 2.4 Confocal Imaging

Confocal imaging experiments were performed using an upright Zeiss LSM 710 NLO confocal microscope (Carl Zeiss). The 405 and 488 nm laser excitation wavelengths were used for the experiments. 10X (NA = 0.3), 20X (NA = 1.0), and 63X (NA = 1.0) W-Plan Apochromat water immersion objectives were used to acquire the 2D images and 3D *z*-stacks. An automatic *z*-compensation of the laser power was applied to have a homogeneous imaging of the sections of the 3D scaffold at different heights. The samples were immersed in PBS at room temperature for the whole duration of the experiments. The images were recorded using Zen (Zeiss) software and analyzed using Fiji^32^ and Imaris (Oxford Instruments, U.K.). An automatic macro in ImageJ was used to identify and count the foci formed within the nuclei of the cells (Figure S2).

### 2.5 Scanning Electron Microscopy (SEM)

SEM images were acquired by a JEOL-JSM 6010LA instrument using 7–20 kV acceleration voltage. The samples were covered with a uniform 13 nm layer of gold sputtered from multiple directions to cover both flat and slanted structures, using a JEOL JFC-1300 Auto Fine coater.

### 2.6 Proton Radiation Experiment Configuration

The layout of the experimental setup and *x*-, *y-*, *and z*-axis configuration at the R&D beamline of the Holland Proton Therapy Center (HollandPTC) are reported in Figure S3. The proton radiation experiments were carried out in the spread-out Bragg peak (SOBP) region^33^ (Figure S4) of the proton beam dose–deposition curve along the *Z*-axis. The Bragg peak is where the maximum proton beam energy is deposited and the SOBP is achieved by the use of a 2D energy modulator,^34^ which creates a plateau characterized by a maximum dose with a uniformity of 98%. A SOBP with a width of 2.5 cm was achieved during the experiments (Figure S4). The beam is passively scattered^34^ in the *X*–*Y* plane (Figure S5) to produce a large field of 100 mm × 100 mm with a dose uniformity of 98%. Water equivalent material (Goettingen White Water-RW3 material, PTW GmbH) is used to adjust the depth so that the sample is located in the middle of the SOBP. The R&D proton beamline of HPTC is a fixed horizontal one; therefore, the samples had to be held vertically to guarantee the dose uniformity during irradiation. For this purpose, specific Petri dish holders were 3D printed by the DEMO (Dienst Elektronische en Mechanische Ontwikkeling) Department at TU Delft (Figures S6 and S7). The doses selected for the experiments were 2 and 8 Gy (delivered with a dose rate of 1.5 Gy/min), since they have shown to induce different foci formation in U-251 when tested with X-rays.^35^ A dose of 2 Gy has also been previously used with carbon ions and shown distinct foci formation in GBM cells.^29^

### 2.7 Mechanical Characterization of the Biomaterial

Pedestals of 50 μm thickness and 250 μm x 250 μm area were employed to evaluate the Young’s Modulus of the IP-Visio material by measuring its stiffness. The compression test was carried out using the FT-NMT03 (Femtotools AG) testing system. It employs a square compression tip of 50 μm x 50 μm, which is indented onto the samples to a depth of 5 μm. From the load/displacement curves, it is possible to extract the stiffness and consequently the Young’s modulus. Figure S8 shows a setup of the schematic for this test.

## 3 Results and Discussion

### 3.1 Design and Fabrication of 3D-Engineered Microenvironments

**Figure 1:**
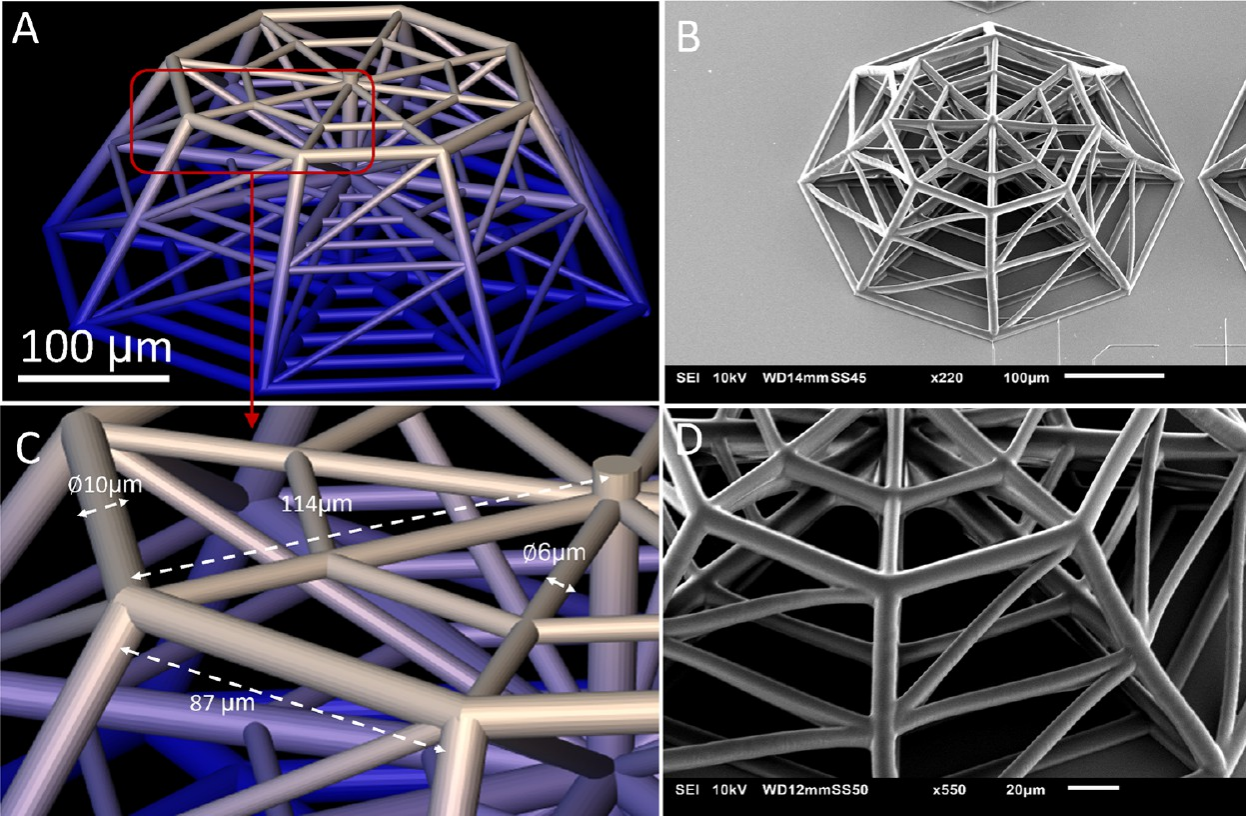
(A) CAD design of the 3D scaffold; (B) SEM image of the 3D scaffold; (C) close-up on the beams and edges of the top layer indicating dimensions; and (D) SEM close-up on the structures of the scaffold.

**Figure 2:**
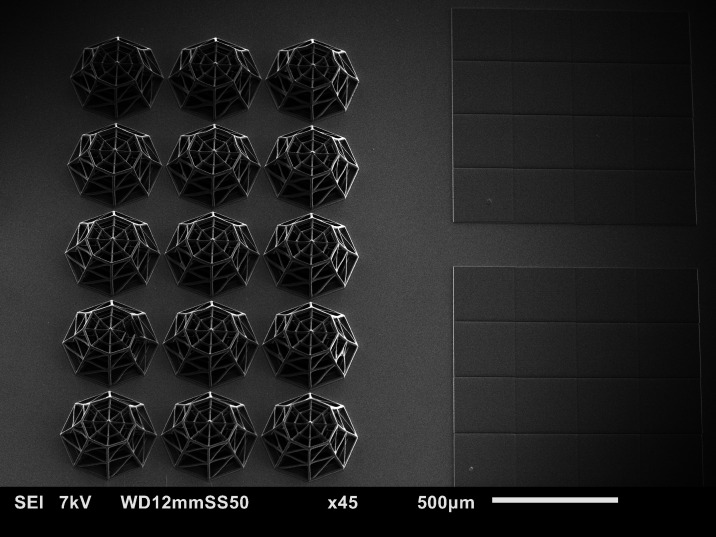
Final sample configuration. Each sample contains 15 3D scaffolds and 3 2D pedestals (here only two are visible).

Figure 1A,B illustrates the overall architecture of the 3D-engineered microenvironment, which fabrication protocol is reported in detail in the Experimental Section section. The architecture of the conceived 3D scaffold (featuring a height of 150 μm) includes an octagonal base (inscribed in a circle of 380 μm diameter). The top layer is similar to the bottom structure scaled down to 0.6 times its size to create the frustum of an octagonal pyramid. The middle layer is located at 70 μm from the base. The lateral horizontal and inclined beams in the structures are created to provide regions of adhesion for cells as well as to ensure the mechanical stability of the architecture. The beams of the mainframe have a diameter of 10 μm, while the other lateral and inclined beams are 6 μm in diameter (Figure 1C,D). The brain vascular branching points have shown to form nutrient-rich and structurally stable regions toward which glioma cells migrate, cluster, and proliferate.^36^ The scaffolds are modeled to replicate these nodes by having beams in multiple planes emerging from central points. The aim of using a pyramidal structure is to create a variation in the size and length of the lateral beams within each scaffold. The beams feature a cylindrical cross section to further provide a mimicry of the blood vessels.^37^ IP-Visio is optimized for the printing of stitch-free structures, each fitting within the writing field of the 25X objective. Independent structures were printed in an array to avoid the possibility of damage due to small bubbles in the resin, incomplete adhesion, and minor printing deformations. The array also allowed us to visualize a quantitatively relevant number of cells. Earlier designs featured a whole pyramid (Figure S9) but were replaced to strengthen the mechanical properties and provide better SEM/confocal imaging access. The final design also had beam diameters and pore sizes comparable to the dimensions of the U-251 cells and could enable adequate colonization of the scaffolds. Each sample includes 15 scaffolds and 3 pedestals of 1000 μm × 1000 μm to provide areas for 2D cell culture on the same material as shown by the SEM micrograph in Figure 2. IP-Visio is a material with negligible autofluorescence, which is a particular advantage for confocal imaging, as the substrate does not interfere with the cell staining used for confocal microscopy (Figure S10). Recent works also reported the fabrication of potentially nonautofluorescent polymeric structures, based either on photoinitiator-free materials^38,39^ or postpolymerization processes.^40^ Further, IP-Visio is noncytotoxic as per ISO-10993-5, therefore biocompatible and appropriate for cell culture applications. In view of this, IP-Visio did not require any additional functionalization or biochemical coating (e.g., laminin, gelatin, fibronectin) to improve the adhesion of the cells on the structures.

To evaluate the Young’s modulus of IP-Visio, a compression test was conducted on 2D pedestals (see Section 2). The compression test yielded an average Young’s Modulus (E) of 1.31 GPa (measured on 10 different pedestals), almost 50 times lower than the Young’s modulus of conventional soda-lime glass substrates (*E* ≈ 70 GPa). Although the intrinsic Young’s modulus of IP-Visio is still far from the one of the brain ECM (0.1–1 kPa^41^), it has a lower value compared to glass and plastic substrates conventionally used in cell cultures and provides a 3D environment that fosteres cell–cell, cell–scaffold interactions and very clear differences in cellular morphologies, as discussed in the following sections.

**Figure 3:**
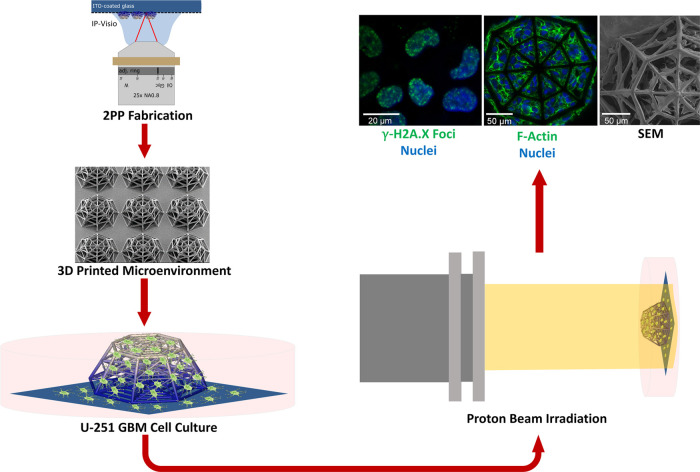
Overview of the experimental process. The samples are fabricated by 2PP, cultured with GBM cells, irradiated with the proton beam, and characterized using SEM and immunofluorescence imaging.

The general overview of the experimental approach developed in the framework of this study is depicted in Figure 3. After fabrication, the samples were sterilized and the U-251 cells were cultured (Section 2) for 5 days. The samples were then either characterized by SEM and immunofluorescence imaging or exposed to proton beam irradiation. In the second case, after radioactive decay of 1–2 h, the samples underwent fixations for SEM and confocal microscopy. Each experiment sample set included one control and two irradiated samples (2 and 8 Gy). Confocal microscopy was used to identify the morphology of the cells and the extent of DNA double-strand breakage, while SEM imaging provided additional high-resolution details on the 3D features of the cell localization and morphology in the scaffolds.

The 3D scaffolds in each sample were printed next to an array of 2D pedestals to simultaneously compare the cells on 2D substrates and 3D microenvironments. IP-Visio pedestals allowed ascertaining that the difference in 2D and 3D morphology was not a result of chemical interaction with the material. The amount of DNA double-strand breakage (DSB) and cell morphology configurations were both compared between 2D and 3D in the presence of different radiation doses. The use of truncated pyramids was essential for confocal imaging as the images were clearest at the top layer, while the “sharp” (Figure S9) cone would trap fewer cells than the grid. The designed 3D architecture allowed analyzing a quantitatively relevant number of cells via confocal imaging. The use of HMDS in the dehydration protocol for SEM sample preparation significantly improved the level of detail visible in the cells and provided deeper insight into the cell morphologies associated with the scaffolds compared to other protocols (critical point drying and sole ethanol, Figure S11). The designed scaffolds were stable and robust enough to withstand changes in medium, stress induced by the cells, and dehydration steps without undergoing any significant change in terms of structure or delamination from the substrate.

### 3.2 SEM and Immunofluorescence Morphological Characterization of U-251 Cells in 3D-Engineered Microenvironments and 2D Pedestals

**Figure 4:**
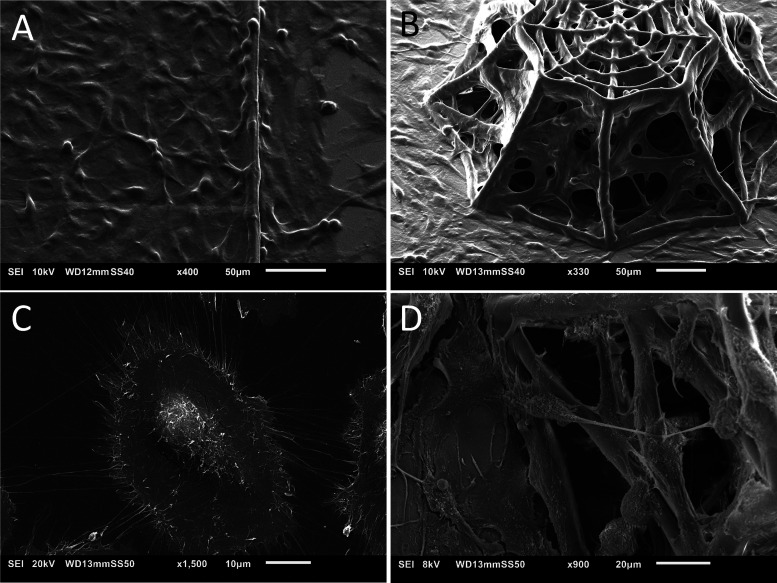
(A) SEM micrograph of the 2D IP-Visio pedestal densely colonized by U-251 cells; (B) SEM micrograph of U-251 cells colonizing 3D scaffolds; (C) SEM close-up micrograph of GBM cells on 2D IP-Visio pedestals with large spread-out morphologies, and small processes; (D) SEM close-up micrograph of GBM cells adhering to the 3D scaffold and forming long processes that extend across the structures.

SEM and immunofluorescence imaging of phalloidin–Hoechst were employed to visualize and compare the morphology of the cells on 2D pedestals and 3D microenvironments as well as to assess the biomaterial–cell interactions. Figure 4 shows an overview of GBM cell colonization on both 2D IP-Visio pedestals and 3D IP-Visio-engineered microenvironments. On 2D pedestals (Figure 4A,C), the morphology of the cells was spread-out, leading to the formation of conventional cell monolayers and occupied a much larger surface area compared to cells growing in 3D microenvironments (Figure 4B).

In the 3D-engineered microenvironments, the cells often assumed a more spherical morphology and colonized the inner core as well as the outer shell of the architecture, displaying a cytoskeletal configuration able to evolve along the *x-*, *y-*, *z-*axis by forming long processes extending from the cells in the directions of the beams of the scaffolds (Figure 4D). These processes are a characteristic of GBM cells and play an important role in cell migration and invasion.^42,43^

**Figure 5:**
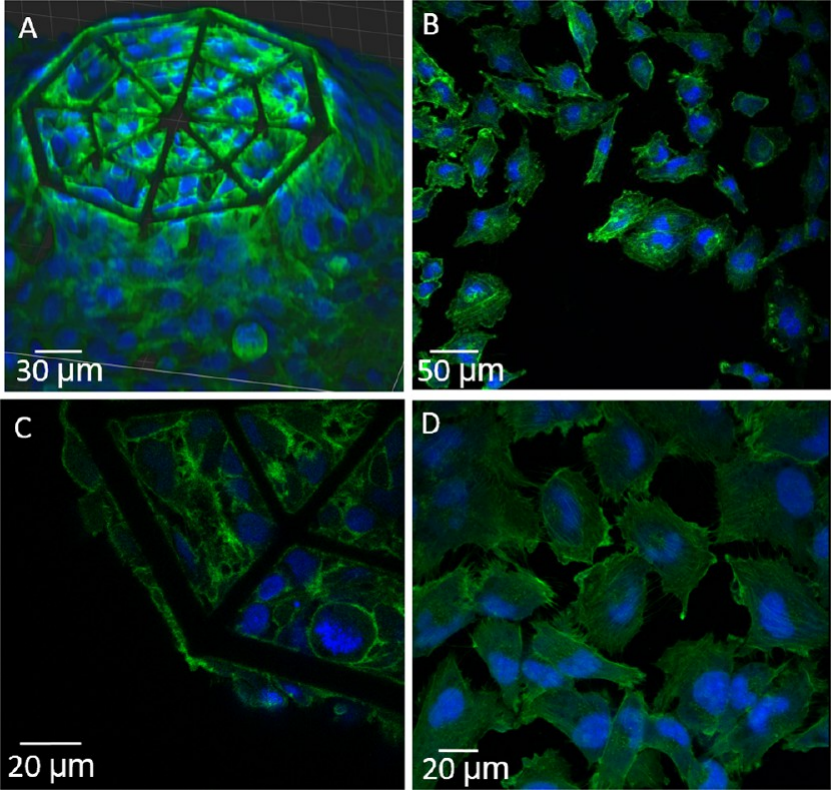
Confocal images of GBM cells in 2D- and 3D-engineered microenvironments. (A,C) Large-view 3D reconstruction and close-up of GBM cells in the 3D IP-Visio scaffold and (B,D) large and close-up view of GBM cells on 2D IP-Visio pedestals (green: phalloidin–F-actin, blue: Hoechst–nuclei).

Although beyond the scope of this study, in future work, it would be interesting to assess, by employing dedicated protein markers (e.g., growth-associated protein 43, GAP-43), if these long protrusions are associated to the generation of tumor microtubes known to have a direct role in the malignancy of GBM.^43^ The cells adhered to and colonized the scaffold efficiently and their growth was “guided” especially next to the nodes (Figure S12), which is also what is observed with blood vessels in the study by Farin et al.^36^ On the other hand, on 2D pedestals, the cells spread out and their processes could not be seen to extend along any specific orientation. Figure 5 shows the F-actin distribution of cells in 2D and 3D configuration. The 3D confocal reconstructions of phalloidin/F-actin-Hoechst/nuclei also give an insightful qualitative indication of the nuclear localization within the cell, as well as its shape and size. From a quantitative point of view, GBM cells grown on 2D IP-Visio substrates featured an average nuclear area of 568.4 μm^2^, 4 times higher than the ones colonizing the 3D IP-Visio microenvironments (139.4 μm^2^). The same trend was observed concerning the cell cytoskeleton, where we observed an average surface area of 2828.9 and 1226.1 μm^2^, respectively, for GBM cells on 2D and 3D environments, which is linked to the flattened, elongated morphology typical of adherent GBM cells in 2D culture.^44^ Finally, the large processes of 3D GBM cells featured an average length and diameter, respectively, of 26.1 and 1.2 μm (aspect ratio equal to 21.7). On the other hand, the thin processes of 2D GBM cells were characterized by an average length and diameter of 12.5 and 0.4 μm, respectively (aspect ratio equal to 31.2).

**Figure 6:**
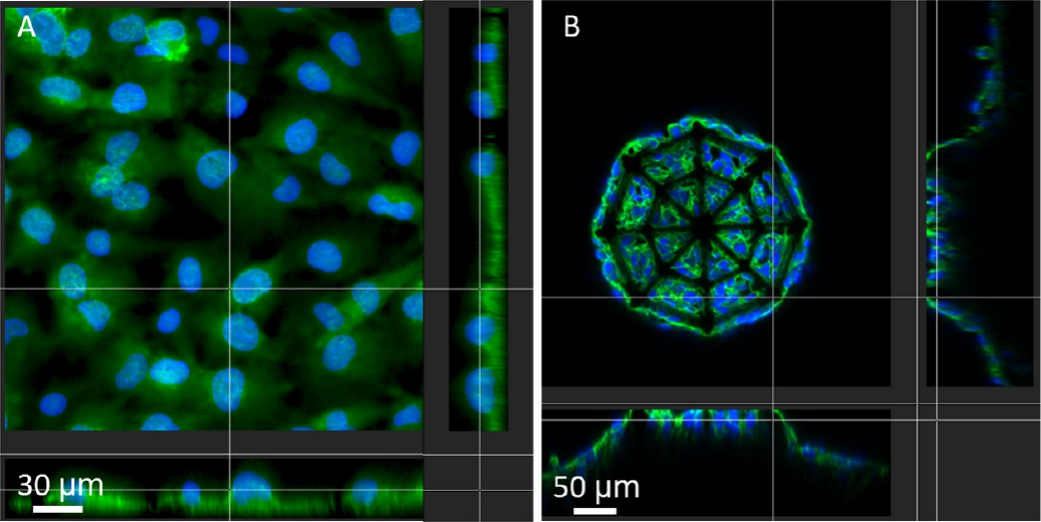
(A) Sectional view of the cells on 2D pedestals and (B) sectional view of the cells in 3D scaffolds (green: phalloidin–F-actin, blue: Hoechst–nuclei).

Figure 6A shows a sectional view of the cells grown on the 2D IP-Visio pedestal and highlights the arrangement of the nucleus as well as the cytoskeleton of the cell. Interestingly, the nuclei are mostly localized in the upper region of the cytoskeleton surface and maintained a spherical morphology. It is known that during migration, the shapes of both the cytoplasm and nucleus adjust according to the environment. In particular, on a 2D substrate, the nucleus can be subjected to tensional forces emanating from the stress fibers and compressive forces due to the actin cap structures and the resistance of the surface.^45^ In 3D scaffolds (Figure 6B), on the other hand, nuclei appear surrounded by the cytoplasm and localized in the inner core of the cytoskeletal envelope. This is in line with the fact that in a 3D environment, the cell migration process requires reshaping of the nucleus as well as of the cytoskeletal envelope to migrate through the pores of the scaffold as it also happens in the openings of the natural extracellular matrix (ECM). The 3D confocal reconstructions of the entire scaffold, although affected by some distortions due to a shadowing effect of the other layers of cells and biomaterial, reported also the presence of GBM cells in the inner core of the 3D microenvironment, showing their ability to invade the whole architecture (Figure S13).

The SEM and confocal images of the GBM cellular morphology in 3D-engineered microenvironments correlate with those observed in earlier publications,^46^ with the cells showing relatively spherical morphology, spindle-like processes, featuring numerous long extensions anchoring the cells across the scaffold beams. GBM cells cultured on 2D pedestals, on the other hand, feature the formation of cell monolayers with a flat cell cytoskeleton and spreading into larger surface areas. The spherical nature of the cells in the 3D microenvironment better resembles the cellular morphology *in vivo*.^46^ The SEM images also corroborate earlier observation reporting how cells grown in 3D environments have a more controlled growth and show lower rates of proliferation.^46^ In the 3D environment, many cells were observed at the nodes of the structure, along the inclined beams and stretched in between the lateral beams. The cells exploited therefore 3D structures as guiding pathways for migration. The direction of their movement cannot be determined without a time-lapse study, but it is known that GBM cells migrate along blood vessel architectures.^36^ This observation can therefore justify further studies focusing on the migratory behavior of the GBM cells in 3D-engineered microenvironments.

### 3.3 Evaluation of DNA Damage of U-251 Cells in 3D-Engineered Microenvironments and 2D Pedestals upon Proton Irradiation

**Figure 7:**
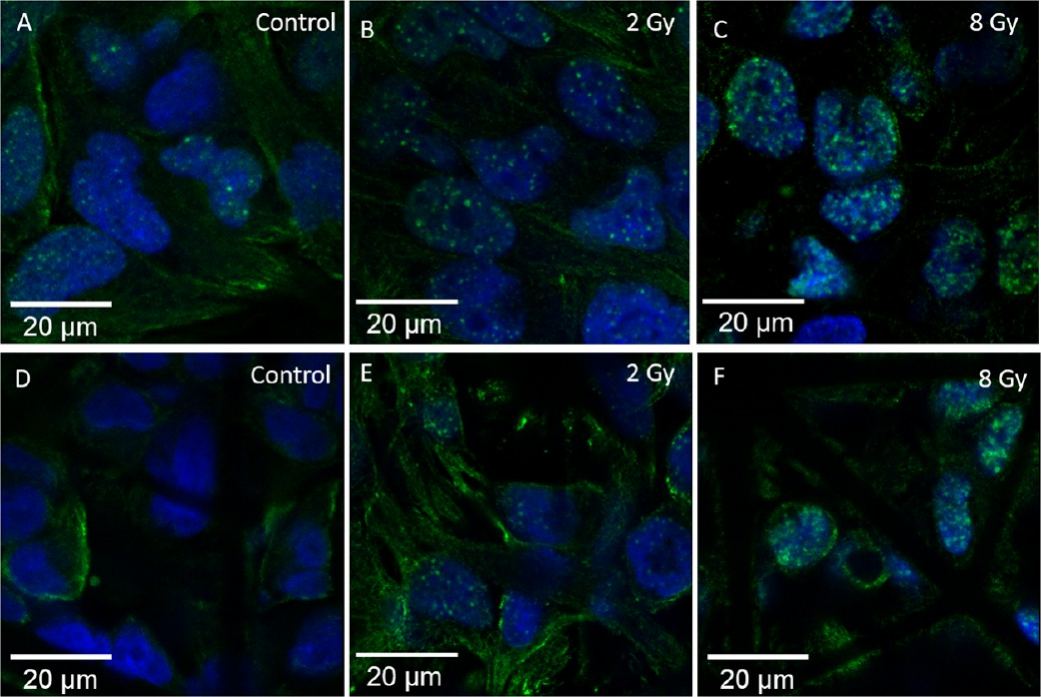
U-251 cells on 2D pedestals and 3D scaffolds after exposure to different doses of proton radiation. (A–C) Cells on 2D pedestals. (D–F) Cells in the 3D scaffold. (A,D) Control samples. (B,E) 2 Gy samples. (C,F) 8 Gy Samples. The number and density of the foci distinctly increase with the increase in dose. Cells in the 2D configuration have more foci than their 3D counterparts. The control sample in (A) also shows some foci, since DSB damage is not exclusively caused by radiation. The images are taken with a 63× magnification lens (green: Gamma H2A.X, blue: nuclei).

Gamma H2A.X foci formation is directly proportional to the extent of double-strand DNA damage in a cell and is also used as a senescence biomarker.^47^ The event of double-strand breakage (DSB) may not occur exclusively by ionizing radiation, but this increases the amount of DSB in cells. The anti-Gamma H2A.X antibody is an established marker to study the formation of such foci for radiation-induced DSB.^48−50^ This marker has also been specifically studied for the U-251 cells^35,51^ employed in this study. In our experiments, Gamma H2A.X foci formation clearly depended on the proton irradiation dose with cells exposed to a greater dose showing higher foci formation. The samples were studied upon 2 and 8 Gy proton beam radiation doses. Gamma H2A.X foci visibility reaches a maximum at 1 h after irradiation and then starts to decrease.^29^ The samples also showed some unintended nonspecific staining of the cell cytoplasm. This may occur when the antibody binds to nonspecific sites in the cells or due to a combination of other ionic and hydrophobic interactions.^52^ The amount of nonspecific staining seemed to decrease in regions of the samples where the cells were sparser. A blocking step was carried out to further reduce such nonspecific staining, although it was still possible to visualize it in some regions. That being mentioned, the foci that were formed are much brighter than the above-mentioned undesired background staining. Additionally, concerning the quantitative analysis, the Fiji^32^ macro only counts the bright spots as local maxima within the nuclear regions in the images, and the effect of nonspecific staining in the analysis is thus avoided. The samples showed a clear difference in the number of foci formed within the nuclei of cells grown on the 2D IP-Visio pedestals and within the 3D IP-Visio engineered scaffolds. An overview of the DNA damage foci after irradiation is depicted in Figure 7. The figure shows a confocal imaging comparison of the foci formation between control, 2 and 8 Gy-irradiated samples, in both 2D and 3D configurations. The control sample is important for every experiment since the Gamma H2A.X foci formation is not exclusively linked to radiation-induced DSBs. The control sample therefore includes all of the foci due to other stresses that the cells undergo and makes the conditions of each experiment more comparable.

**Figure 8:**
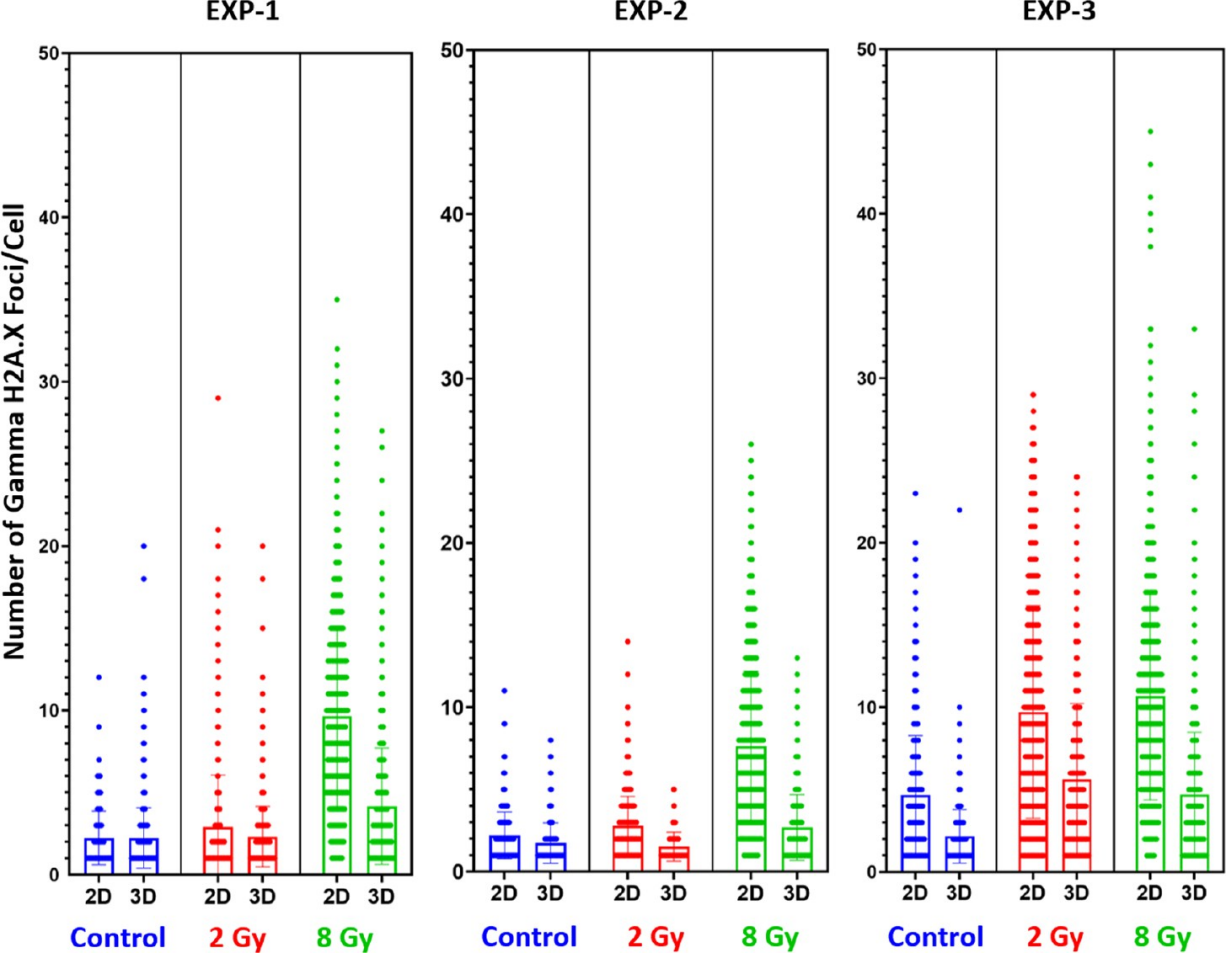
Each graph shows the scatter distribution of the foci formed. The longer width of the horizontal bar represents a greater number of cells with that number of discrete foci. The data correlates for all three experiments and follows a similar trend. The bar chart shows the mean, with error bars.

**Figure 9:**
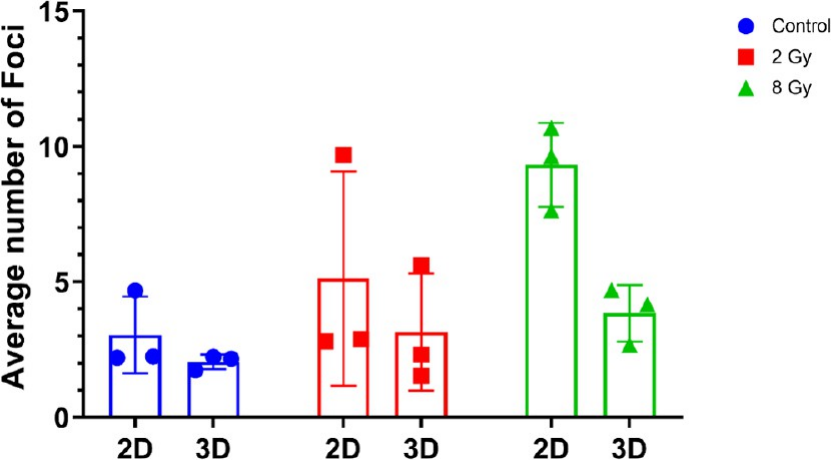
Mean values of each experiment shown in Figure 8. The mean values of the foci increase with an increasing dose even when averaged across multiple experiments. The mean number of foci formed is always lower in 3D scaffolds cells than 2D monolayers.

For all of the conducted experiments, the GBM cells cultured on 3D microenvironments qualitatively and quantitatively showed less foci than the cells cultured on 2D pedestals. Additionally, the results showed that the percentage of cells positive for Gamma H2A.X foci upon 8 and 2 Gy irradiation are, respectively, 10 and 20% higher in 2D as compared to 3D. These experiments were repeated thrice on different dates and displayed an equivalent trend. This can be seen quantitatively in Figures 8 and 9, where we report the number of Gamma H2A.X foci per cell in 2D or 3D microenvironment configuration and under three different conditions (control–nonirradiated, 2, 8 Gy).

Finally, 2D pedestals and 3D-engineered GBM microenvironments were also compared in terms of SEM morphology between the control and proton-irradiated samples. Figure S14 shows a comparison between the control and 8 Gy-irradiated samples fixed 12 h after proton irradiation. There were no observed differences between the morphologies of the cells. We also underline that some dead or apoptotic cells, featuring low adherence to the material,^53^ might have been removed during the fixation and dehydration steps required for the SEM characterization.

It has been established that the ECM plays an important role in the regulation of proteins and other markers for GBM cells, and this induces changes to their morphology, cell–cell interaction, and cell–matrix interaction.^54,55^ We hypothesize that the observed difference in the number of foci is directly correlated with the differences in terms of cytoskeletal properties and cell–matrix interactions in 2D and 3D cell culture configurations. One example is the position of the nucleus for cells grown in 2D, mostly localized in the upper region of the cytoskeleton surface, while for cells in 3D, localized in the inner core of the cytoskeletal envelope as mentioned in Section 3.2. This observation is noteworthy in light of recent findings reporting on the role of nuclear mechanics and its impact on DNA damage.^56^ Further, having used a 2D IP-Visio pedestal eliminates the possibility that these differences are caused because of a chemical interaction (with the material) alone. We can therefore infer that the addition of the third dimension and the presence of micrometric beams with a diameter close to the one of brain blood vessels,^57^ trying to better mimic the 3D spatial configuration of the native GBM microenvironment, affects the response to proton irradiation of the cells which show higher radioresistance compared to 2D GBM cell monolayers.

Cell sensing, migration, differentiation, proliferation, apoptosis, gene expression, and signal transduction are all influenced by mechanical stimuli^58,59^ of the surrounding ECM. Hence, a difference in ionizing radiation response between 2D cell monolayers and 3D cell microenvironments is an expected outcome.^60^ The tumor microenvironment and the interaction of cancer cells with the ECM play indeed a very important role in the resistance of cells to treatment.^61^ Previous studies on GBM cells reported how *in vivo* orthotopic xenograft models (in which cell lines or patient-derived cells are transplanted into a host of a different species) showed higher radioresistance than the corresponding *in vitro* cultures.^62^ Further, 3D spheroid cultures featured higher surviving fractions of cells when treated with radiation.^63^ 3D models of other cancers also showed higher radioresistance in 3D.^64^ In their study, Jamal et al. compared the radiation response of a GBM xenograft model to a 2D *in vitro* cell culture model.^65^ They found that the *in vivo* xenograft was characterized by fewer Gamma H2A.X foci formation than 2D cell cultures. An *in vivo* environment can therefore greatly differ from unrealistic 2D cell culture conditions. It can be reasonably assumed that cells in an *in vivo* environment are also growing along tridimensional spatial configurations among other conditions. Considering this, the results of our study correlate with the findings of Jamal et al.^65^ Fewer foci formed in the 3D scaffolds can suggest that, even without other factors (proteins, nutrients, and ECM components), the integration of the third dimension, in the form of structures which try to minimalistically mimic the brain blood vessels^37^ and guide the growth of GBM cells, can make the cells more resistant to proton radiation. Hence, this makes our 3D *in vitro* model an effective benchmark tool to better estimate the response of an *in vivo* model to proton radiation. Another possible explanation of our results is that in 3D cell cultures, the repair dynamics of the cells are different. Many factors can affect the efficiency and extent of DSB repair in GBM cells.^66^ Previous studies have shown indeed that repair kinetics are different in 3D cell models as compared to 2D,^64,67^ therefore suggesting that GBM cells in a 3D environment may also exhibit different DSB repair mechanisms.

## 4 Conclusions

In this work, we reported the design and manufacturing of a biomimetic scaffold to create *in vitro* replicas of the *in vivo* GBM microenvironment. We developed the scaffolds inspired by the blood vessel architecture and its vascular branching points, where GBM cells cluster and proliferate.^36^ The use of 2PP enabled the fabrication of accurate topographies based on the novel biomaterial IP-Visio featuring high biocompatibility and negligible autofluorescence. The use of direct immunofluorescence and scanning electron microscopy showed that U-251 cells efficiently colonized the 3D scaffolds and presented remarkable differences in terms of morphology and nuclear organization compared to cells grown on 2D IP-Visio pedestals. Specifically, cells on the 3D scaffolds showed morphologies closer to those observed *in vivo*. We also reported, for the first time, the proton beam irradiation response of GBM U-251 cells cultured on such biomimetic scaffolds. We observed a clear difference between the control, 2, and 8 Gy-irradiated samples. The extent of DNA damage was quantitatively analyzed by exploiting the Gamma H2A.X antibody. Compared to 2D GBM cell monolayers, GBM cells consistently showed lower damage in 3D-engineered microenvironments, which correlates with the response of GBM cells *in vivo* and other *in vitro* 3D models, where a greater radioresistance is observed.^63,68,69^ The reported protocol provides an *in vitro* benchmark tool for proton radiobiology, which paves the way to better understand GBM cells–proton radiation interactions, decreasing the amount of *in vivo* studies required to assess the radiation response of GBM and, in future, of other cancerous tissues. The following development of this study could include 3D scaffolds able to mimic even better the brain vasculature, by the integration of variable beam thickness and curved geometries closer to the ones of real blood vessels, as well as the use of patient-derived GBM cells. Additionally, we envision a co-culture model with brain vascular endothelial cells (the building blocks of brain blood vessels) to include the biochemical signals of the microvascular architecture and assess the cellular response to irradiation in such an *in vitro* system.

## Supporting Information Available

Additional images and information concerning the experimental setup, proton beam characteristics, quantification methods, mechanical measurements, design iterations, cell morphologies, and protocol development (PDF)

## Abbreviations

2PP: two-photon polymerization
GBM: glioblastoma
NIR: near-infrared
SOBP: spread-out Bragg peak
SEM: scanning electron microscopy
DSB: double-strand breakage
