# Supplementary material for: Evaluation of Proton-Induced DNA Damage in 3D-Engineered Glioblastoma Microenvironments
Source: ACS Appl Mater Interfaces. 2022 Apr 20;14(18):20778–89. doi: 10.1021/acsami.2c03706 (PMC9100514; doi:10.1021/acsami.2c03706)
Supplement: Supplementary file 1 — am2c03706_si_001.pdf [file am2c03706_si_001.pdf]

Supporting Information

Evaluation of proton-induced DNA damage in 3D Engineered Glioblastoma Microenvironments

Qais Akolawala[a], Marta Rovituso[b], Henri H. Versteeg[c], Araci M.R. Rondon[c], Angelo Accardo[a,]*

[a]Department of Precision and Microsystems Engineering, Delft University of Technology, Mekelweg 2, 2628 CD Delft, The Netherlands.

[b]Holland Proton Therapy Center (HollandPTC), Huismansingel 4, 2629 JH Delft, The Netherlands.

[c]Einthoven Laboratory for Vascular and Regenerative Medicine, Division of Thrombosis and Hemostasis, Department of Internal Medicine, Leiden University Medical Center, 2333 ZA Leiden, The Netherlands

**Corresponding author's e-mail address: A.Accardo@tudelft.nl*

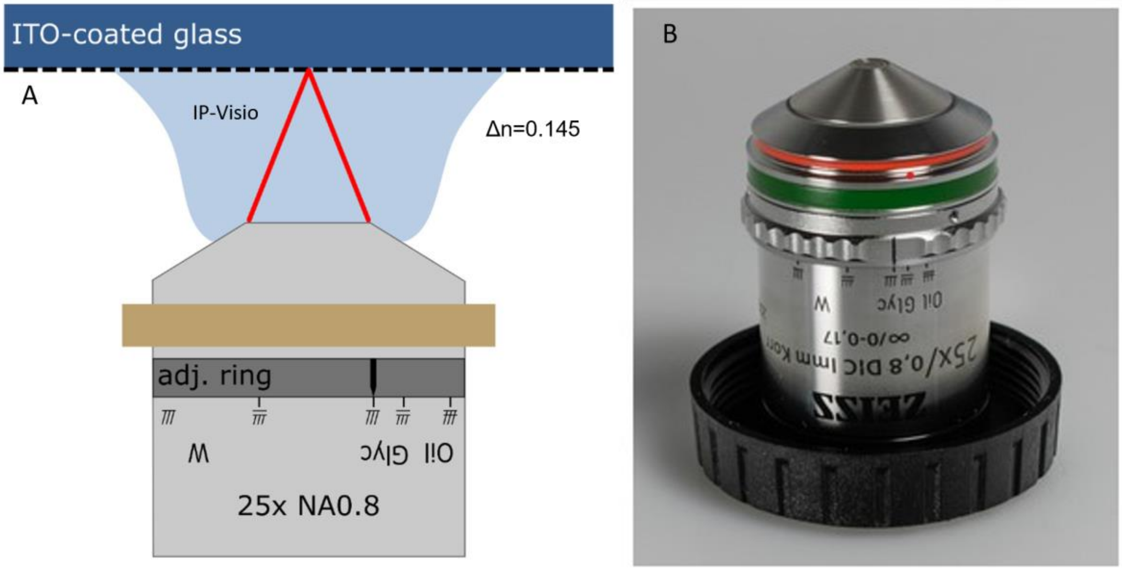

Figure S1. A: Schematic of the DiLL configuration used to manufacture the IP-Visio scaffolds by 2PP; B. Actual image of the immersion 25X objective lens used for 2PP.

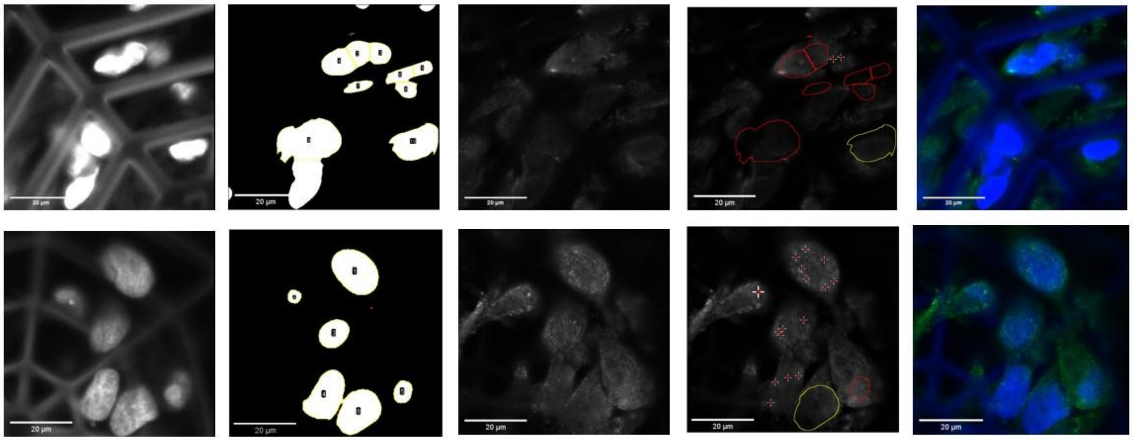

Figure S2. Schematic of the macro used for foci counting. The Macro identifies regions of the nuclei and converts them into a mask. It then counts the bright regions by identifying local maxima only within the area marked by the mask. The output is given as a count/region[1].

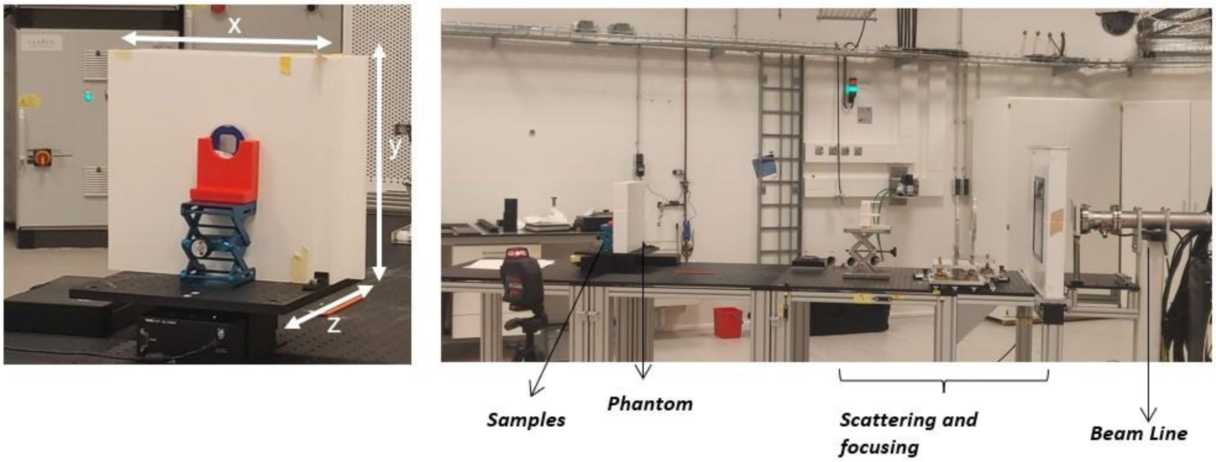

Figure S3. Layout of the experimental set-up at HollandPTC. The phantom are slabs of RW3 material, which is used to align the Spread-out Bragg Peak (SOBP) at the sample to deliver the highest dose. The phantom is used to adjust the beam in the z direction, while the X-Y plane has a uniform dose region of 100 mm X 100 mm.

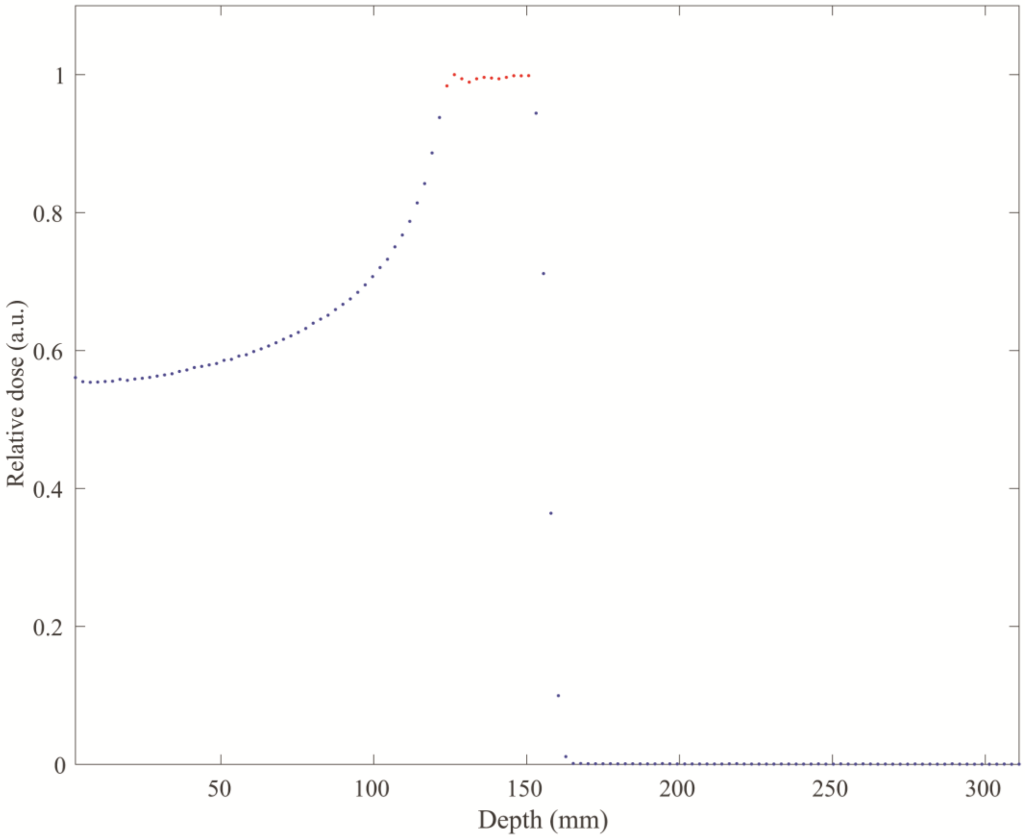

Figure S4. Graphical representation of the actual SOBP employed during the experiments with a uniform region of 2.5 cm in which the samples are irradiated.

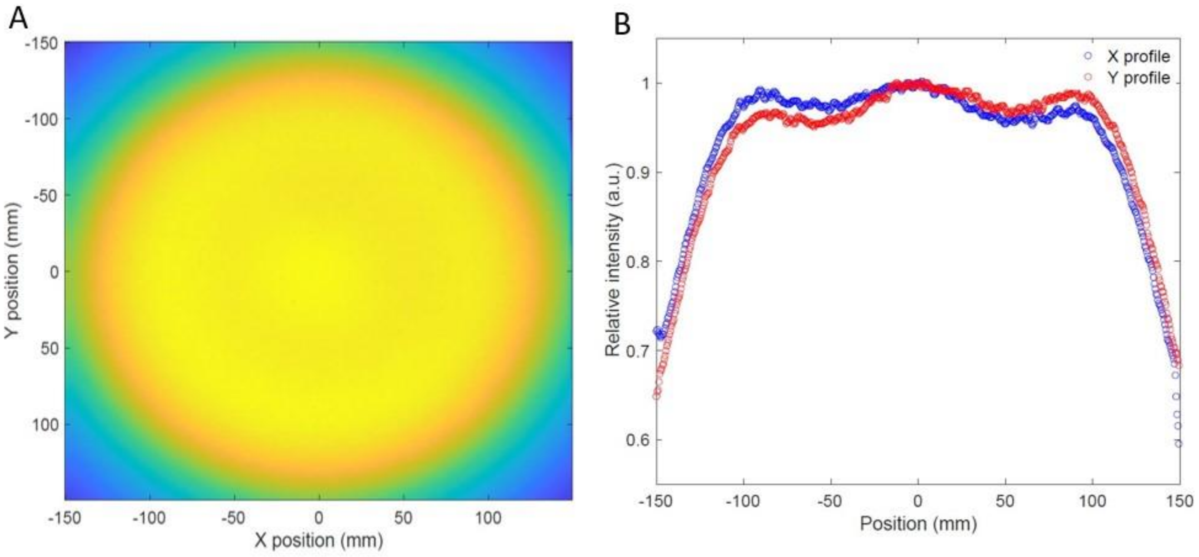

Figure S5. A: Visual representation of the uniform dose region marked in yellow; B: cross sectional profiles of the relative intensities of the regions shown in A.

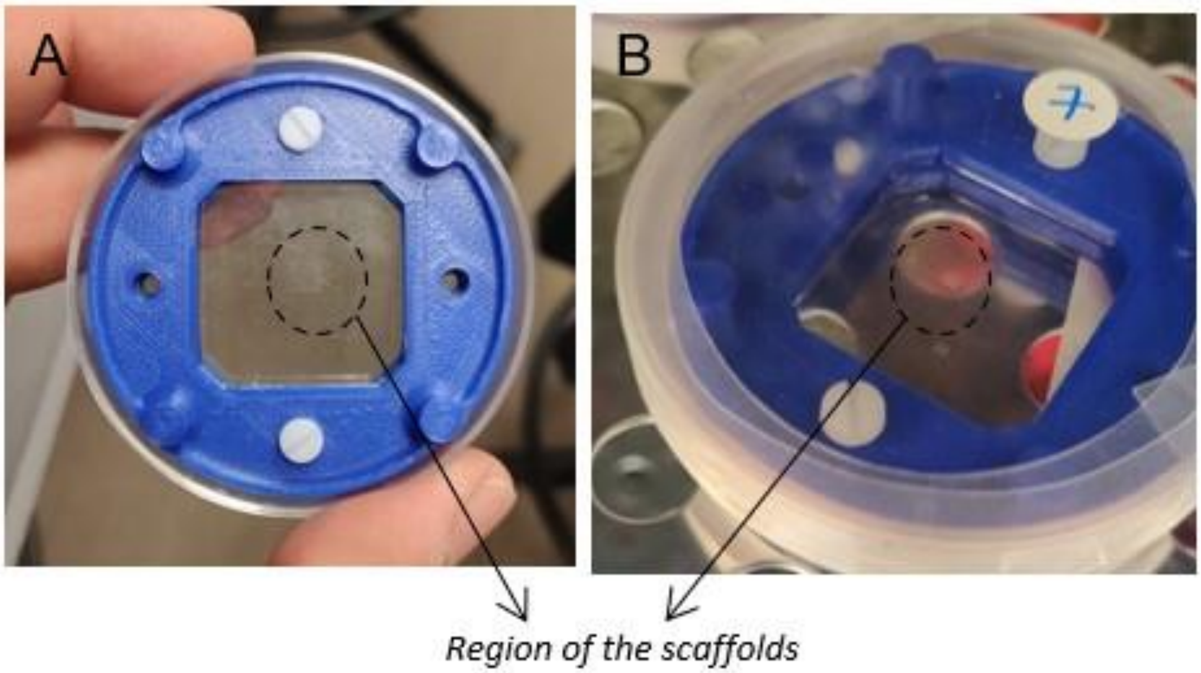

Figure S6. A: The blue insert shown in the image is a 3D printed glass-slide holder that is held together by screws. It holds the glass slide in place when it has to be held vertically and irradiated; B: Sample (with cell medium present) sealed with parafilm and ready to be irradiated.

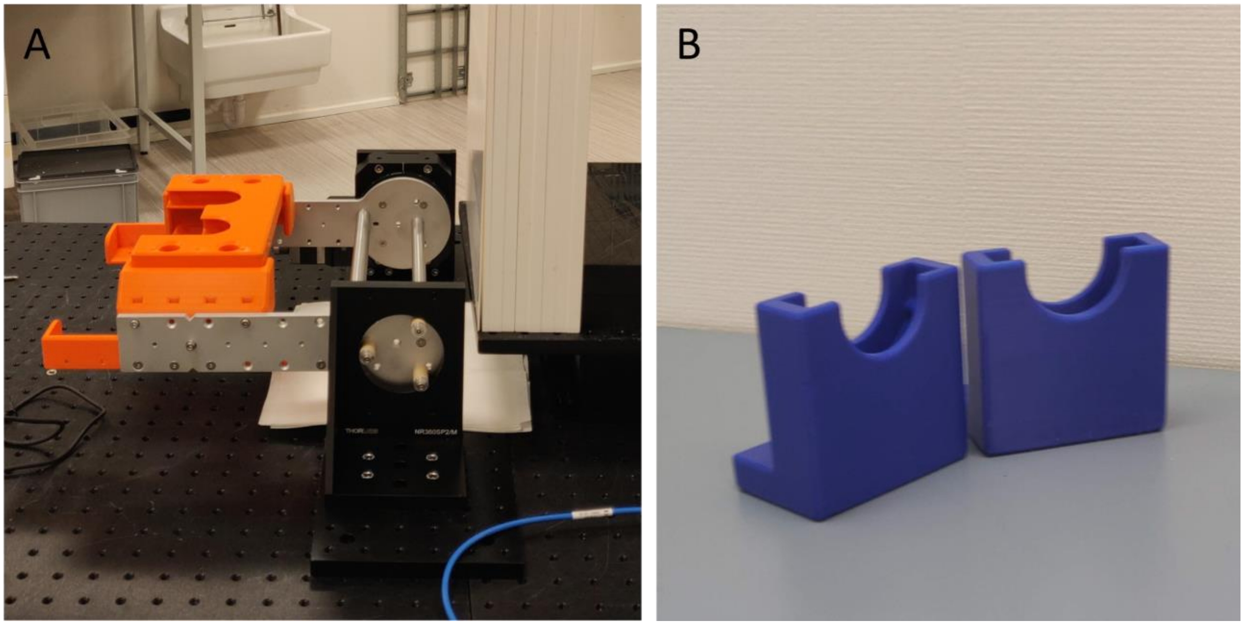

Figure S7. A: automated flipper employed in later experiments to automatically keep the samples vertical and align them correctly; B: sample holders used initially fitting the size of the sealed petri dishes and able hold them in the center of the uniform dose deposition field of the proton beam.

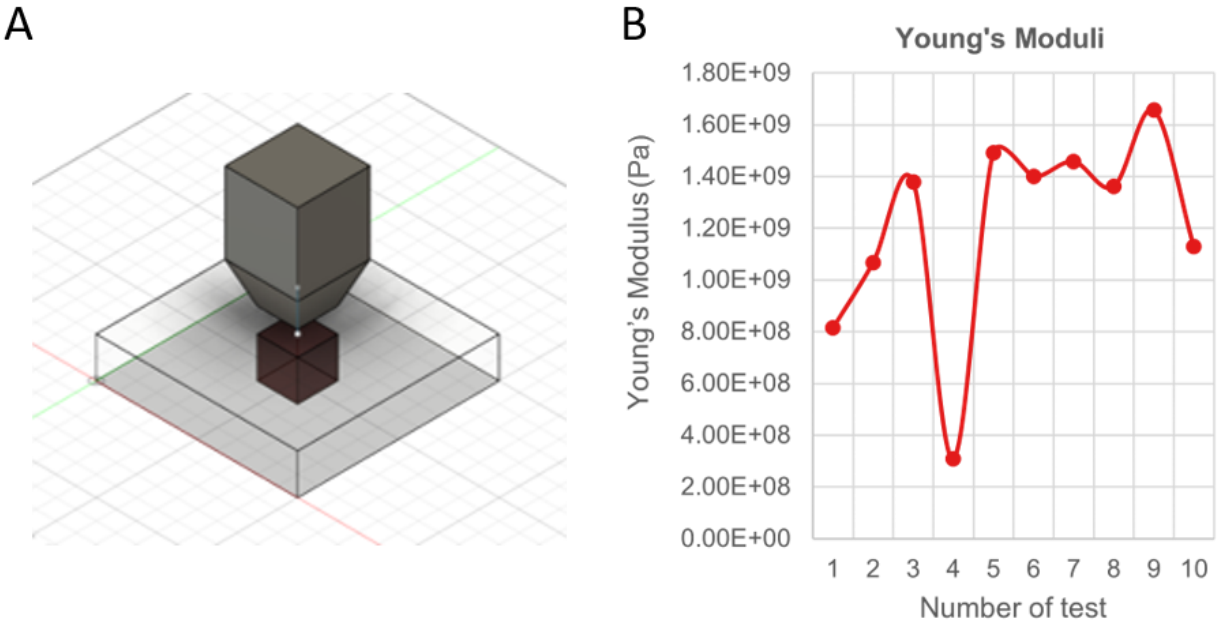

Figure S8. A. Schematic of the probe used to compress the IP-Visio pedestals for the Young's Modulus measurement; B: Graphical representation of the different values for 10 independent measurements. The average value found for the Youngs' modulus is 1.31 GPa, for which the 4th value is considered and outlier possibly caused by the probe not being at the center of the pedestal due to the manual adjustements required with the Femtotools setup.

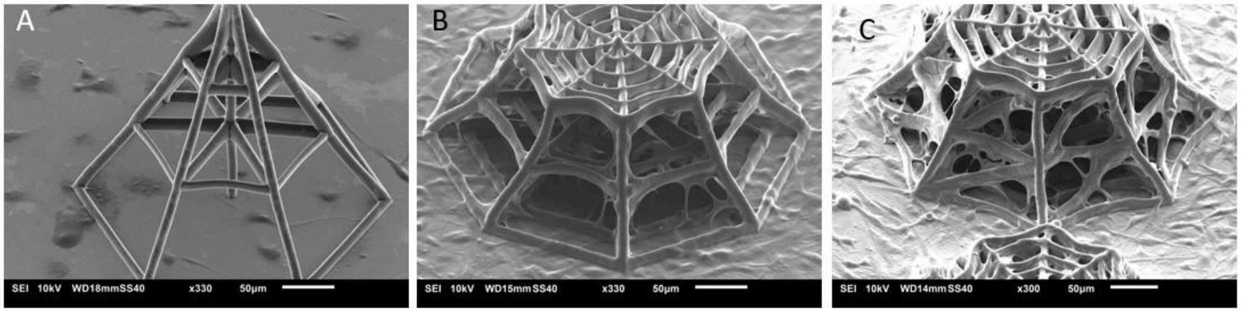

Figure S9. Optimization of cell densities on different scaffold geometries. A. The cells do not adhere/colonize due to the low cell seeding densities and a 3D architecture featuring too large gaps; B. The truncation of the pyramid and addition of lateral beams entraps more cells in the scaffold; C. Optimized cell density of 50,000 cells/ml in the final design with angular beams to make the "windows" in the scaffold smaller. The angular beams also become pathways for cells to migrate.

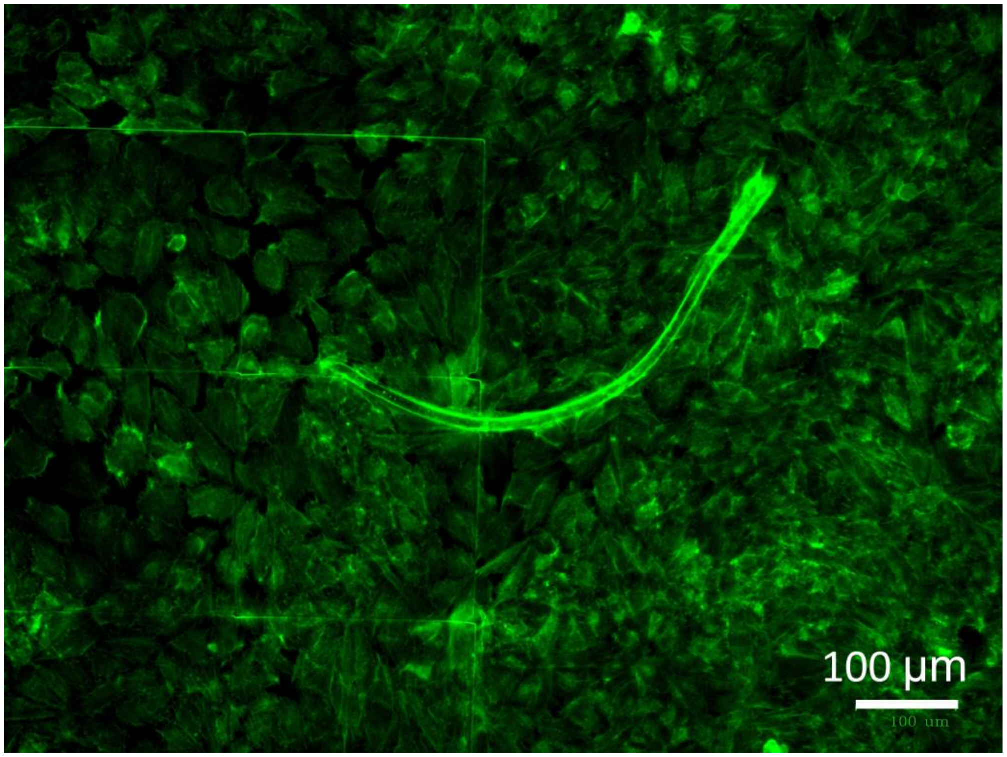

Figure S10. IP-Visio pedestal cultured with GBM cells. The pedestal is not fluorescent, but the edges can be visualized due to the cells growing on them. (Green: Phalloidin-FITC staining the F-Actin filaments in the GBM cells)

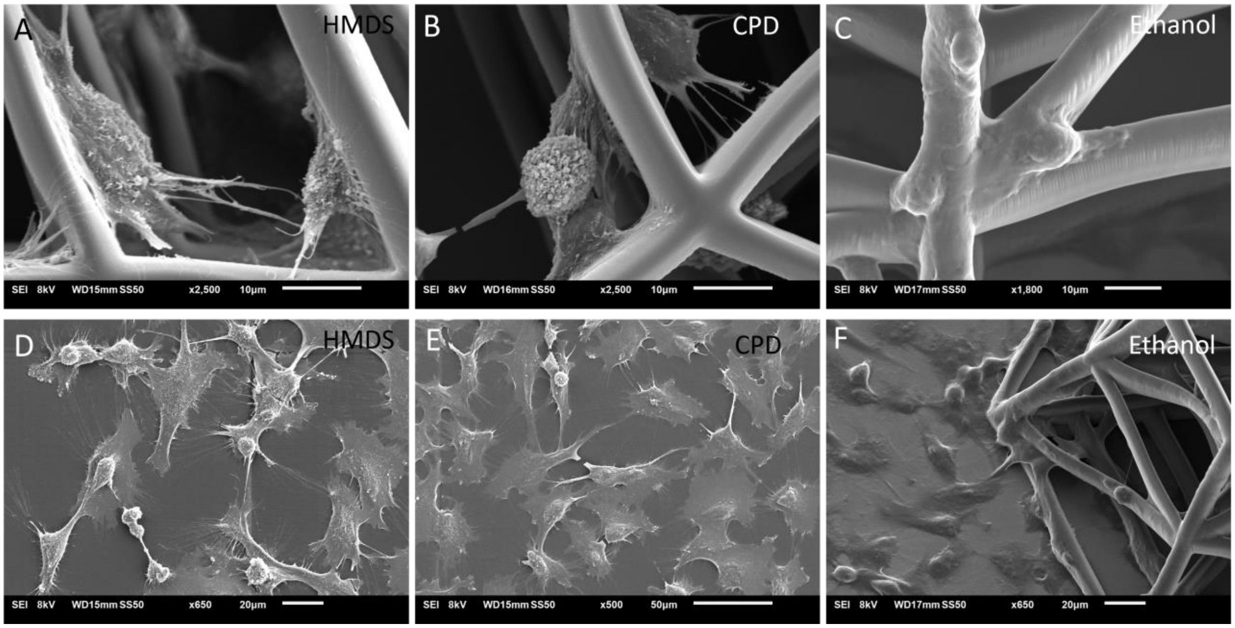

Figure S11. SEM image comparison between 3 dehydration protocols. A,D: The finally selected HMDS protocol; B,E: Critical Point Drying (CPD) protocol which did not improve the details when compared with HMDS; C,F: Ethanol protocol in which the cell surface is not as vivid as the after employing the other methods.

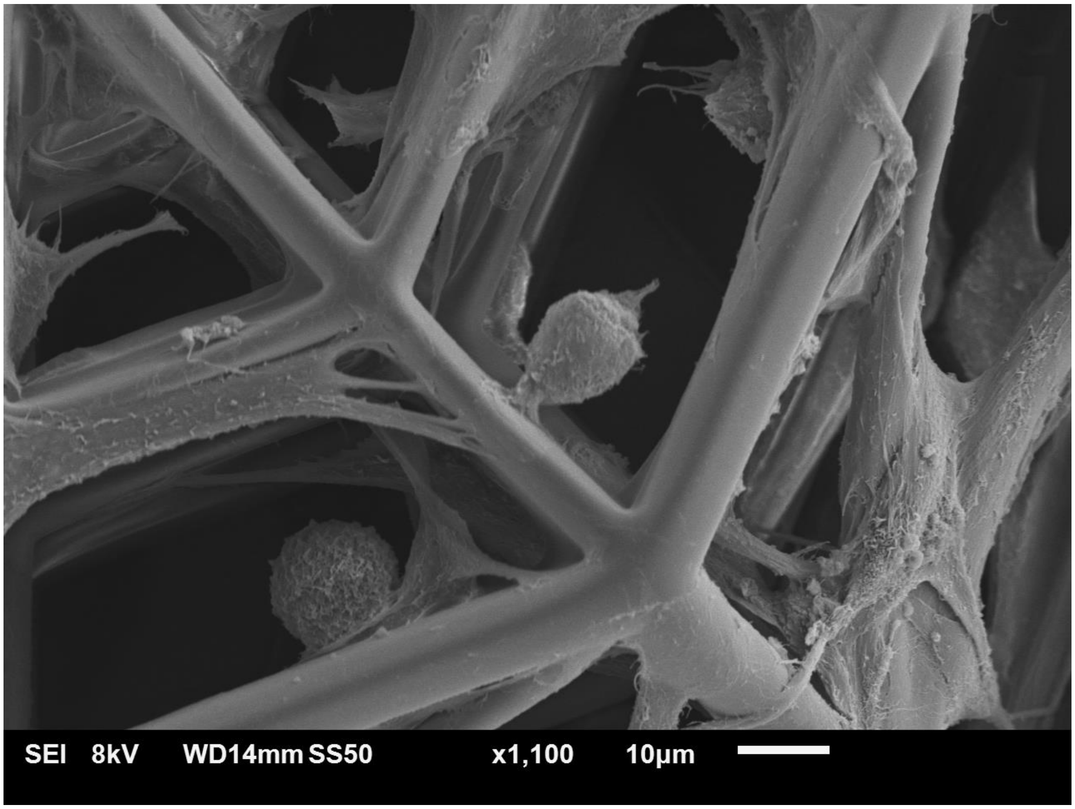

Figure S12. Typical view of the GBM cells on the scaffolds. The cells are present in between lateral beams, on inclined beams, and in the nodes formed by the structures attached to more than one beam.

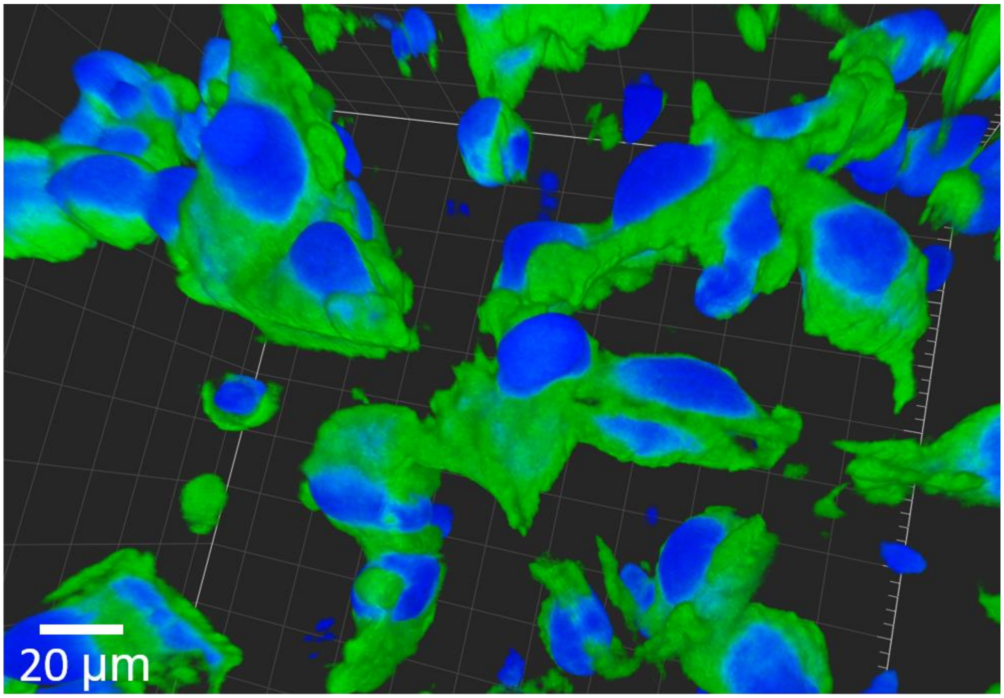

Figure S13. 3D Reconstruction of a layer of cells deep inside the 3D scaffold. The image shows noise and regions of very high fluorescence due to the high laser power leading to some distortions in the reconstruction.

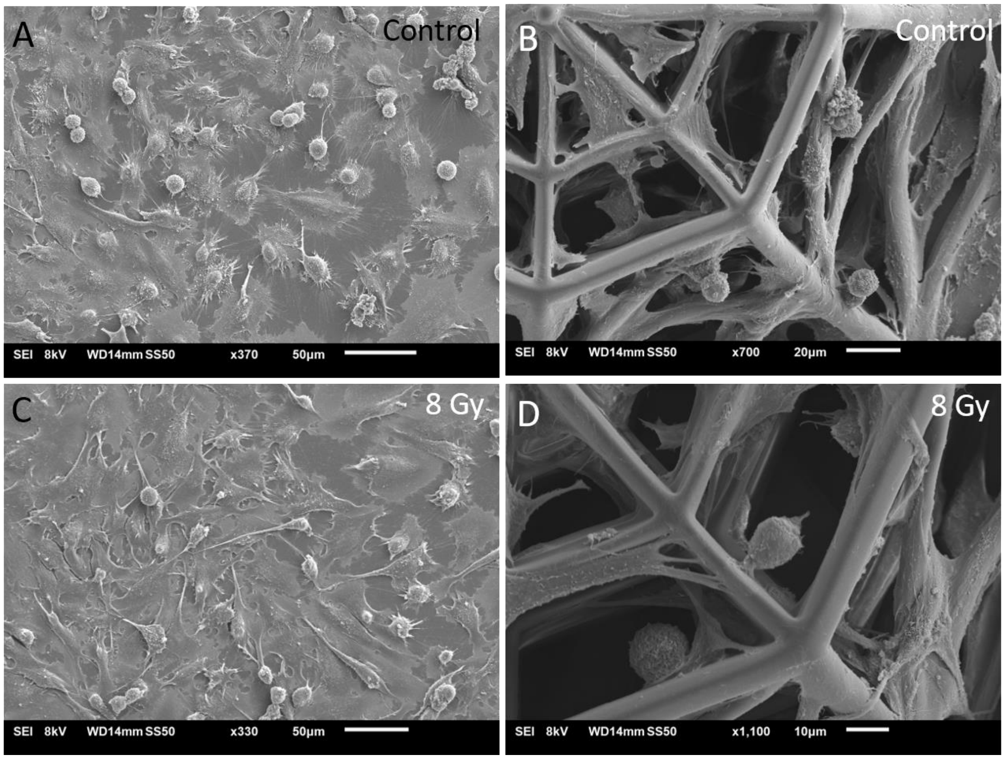

Figure S14. SEM morphological comparison between control (A,B) and 8 Gy proton irradiated samples (C,D).

REFERENCES

(1) Schindelin, J.; Arganda-Carreras, I.; Frise, E.; Kaynig, V.; Longair, M.; Pietzsch, T.; Preibisch, S.; Rueden, C.; Saalfeld, S.; Schmid, B.; Tinevez, J. Y.; White, D. J.; Hartenstein, V.; Eliceiri, K.; Tomancak, P.; Cardona, A. Fiji: An Open-Source Platform for Biological-Image Analysis. *Nature Methods 2012 9:7* **2012**, *9* (7), 676–682. https://doi.org/10.1038/nmeth.2019.

(2) Vitti, E. T.; Parsons, J. L. The Radiobiological Effects of Proton Beam Therapy: Impact on DNA Damage and Repair. *Cancers 2019, Vol. 11, Page 946* **2019**, *11* (7), 946. https://doi.org/10.3390/CANCERS11070946.
